# Diverse nanostructures underlie thin ultra-black scales in butterflies

**DOI:** 10.1038/s41467-020-15033-1

**Published:** 2020-03-10

**Authors:** Alexander L. Davis, H. Frederik Nijhout, Sönke Johnsen

**Affiliations:** Department of Biology, Duke University, Durham, NC 27708 USA

**Keywords:** Nanoscale biophysics, Evolution, Optical materials and structures, Bioinspired materials

## Abstract

Recently, it has been shown that animals such as jumping spiders, birds, and butterflies have evolved ultra-black coloration comparable to the blackest synthetic materials. Of these, certain papilionid butterflies have reflectances approaching 0.2%, resulting from a polydisperse honeycomb structure. It is unknown if other ultra-black butterflies use this mechanism. Here, we examine a phylogenetically diverse set of butterflies and demonstrate that other butterflies employ simpler nanostructures that achieve ultra-black coloration in scales thinner than synthetic alternatives. Using scanning electron microscopy, we find considerable interspecific variation in the geometry of the holes in the structures, and verify with finite-difference time-domain modeling that expanded trabeculae and ridges, found across ultra-black butterflies, reduce reflectance up to 16-fold. Our results demonstrate that butterflies produce ultra-black by creating a sparse material with high surface area to increase absorption and minimize surface reflection. We hypothesize that butterflies use ultra-black to increase the contrast of color signals.

## Abstract (web summary)

Nature has developed the ability to produce a wide range of optical effects most notably in the butterfly wing. Here, the authors report on the analysis of the structures responsible for ultra-black coloration across different butterflies and combine this with modelling to identify the key characteristics

## Introduction

Ultra-black materials, those with both exceptionally low reflectance and high absorbance, have the potential to increase photovoltaic cell efficiency, improve stray light capture in telescopes, and inform the design of infrared or radar camouflage, among other applications^1–4^. Currently, most synthetic ultra-black materials are made from nano-patterned metals or carbon nanotubes^5,6^. In both materials specular reflection is reduced by nano-scale surface roughness caused by either acid etching in the case of nickel-phosphorous alloys or nanotube deposition in carbon arrays. Light that is not reflected from the surface is scattered within the material until it is absorbed. The blackest of these synthetic ultra-black materials (Vantablack) is made from a sparse array of vertically aligned carbon nanotubes^5^. The nanotubes, however, must be fabricated at high temperatures and are extremely susceptible to abrasion and other forms of damage, making them unsuitable for many uses. Naturally occurring ultra-black materials may offer insight into more robust absorbers for future replication.

Recently, it has been shown that several animals have evolved micro- or nanostructures that reflect as little as 0.05% of visible light, even at normal incidence^7–11^. Several species of birds of paradise have evolved complex barbule microstructures that increase light scattering and, consequently, the number of opportunities for light absorption by melanin embedded within the feather^10^. Similarly, certain peacock jumping spiders have evolved a cuticular micro-lens array that limits surface reflection with multiple scattering between adjacent lenses and allows the light to pass further into the cuticle where it is absorbed^11^. Lastly, certain papilionid butterflies produce ultra-black wing patches with two layers of thin (~2.5 µm), overlapping scales that have an upper lamina patterned with a quasi-honeycomb structure made of crossribs connecting ridges^8,9^. Butterflies, in particular, offer a versatile study system for investigating natural ultra-black surfaces because: (i) their scales have evolved a number of optical specializations, including multilayer reflectors and thin fims^12^, (ii) the scales are several times thinner than other naturally occuring ultra-black materials or synthetic alternatives, and (iii) butterfly scales are under constraint to be both light and robust for use in flight.

Previous studies have demonstrated the nano-holes in the upper lamina of papilionid butterfly scales allow light to enter the interior of the scale where it is absorbed by melanin that is bound to chitin in the cuticle^8,9,13^. Additionally, slabs patterned with holes approximating the size of those in the butterflies (240 nm) have been shown to increase light absorption when compared to an un-patterned slab^9^. The role of the steep periodic ridges that border these nano-holes is less certain. One modeling effort supports an important role for the ridges in increasing absorption by channeling light into the holes^13^, while another found they have an insignificant effect on broadband absorption—particularly in the visible range^9^. These studies provide a foundation for evaluating how the structure of butterfly scales enhances light absorption, however, they are restricted to the family Papilionidae, they do not examine the differences between regular and ultra-black scales, and they consider absorption instead of reflectance (the latter of which is more relevant in biological contexts, such as signaling or camouflage).

Here, we use spectrophotometry, scanning electron microscopy, and finite-difference time-domain modeling to investigate the nanostructure underlying ultra-black wing patches in butterflies from four subfamilies to derive general design principles of natural ultra-black materials. We find that, despite considerable variation in the size and shape of the nano-holes, all ultra-black scales have steep ridges and trabeculae that are substantially deeper and wider than those found in regular black or brown scales. We confirmed with optical modeling that these two structural elements are the key components in producing low reflectance, and that removing either of them drastically increases the reflectance by over an order of magnitude. Given that these structural changes increase the surface area for absorption, we conclude that butterflies operate under the same design principles as synthetic ultra-black materials—high surface roughness and a large area for absorption. We also hypothesize that because the ultra-black patches always border colored, white, or bright iridescent patches (i.e., there are no known butterflies that are entirely ultra-black) that they are used to increase the perceived brightness and saturation of colors used in aposematic or intraspecific displays.

## Results

### Wing reflectance spectra

**Fig. 1:**
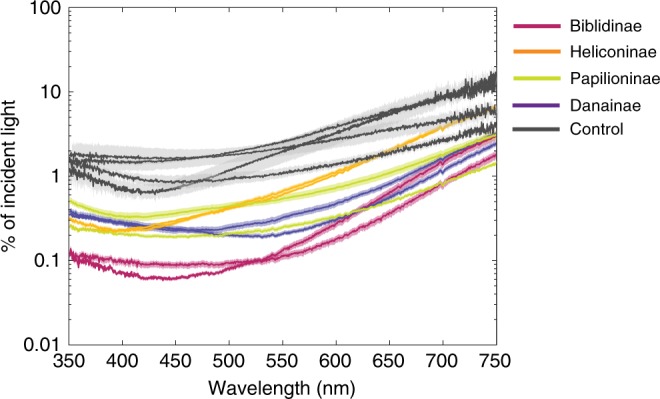
Reflectance of ultra-black and control butterflies. Reflectance (+/− 1 SE; *n* = 3–5 depending on the species) from 11 ultra-black butterflies from five subfamilies. Note the logarithmic scale for reflectance (*y*-axis).

To explore the potential diversity of nanostructures underlying ultra-black color in butterflies, we first selected ten butterfly species visually assessed to be exceptionally black that represent four subfamilies: Papilioninae, Biblidinae, Danainae, and Heliconinae (*n* = 10 species; Supplementary Table 1). We also chose brown or less black butterflies from four genera that contain ultra-black species to serve as controls (*n* = 4; Supplementary Table 1). All of the ultra-black butterflies measured here had reflectances between 0.06% and 0.4% at 500 nm under perpendicular incident illumination (Fig. 1) (approximately the wavelength of minimum reflectance for all species), approaching those of synthetic ultra-black materials like Vantablack (Surrey Nanosystems, New Haven, UK) and surpassing the darkest naturally occuring materials (ultra-black plumage of birds of paradise) when measured under the same geometry (unpublished data). Control butterflies had reflectance values between 1 and 3%.

### Scale nanostructure

**Fig. 2:**
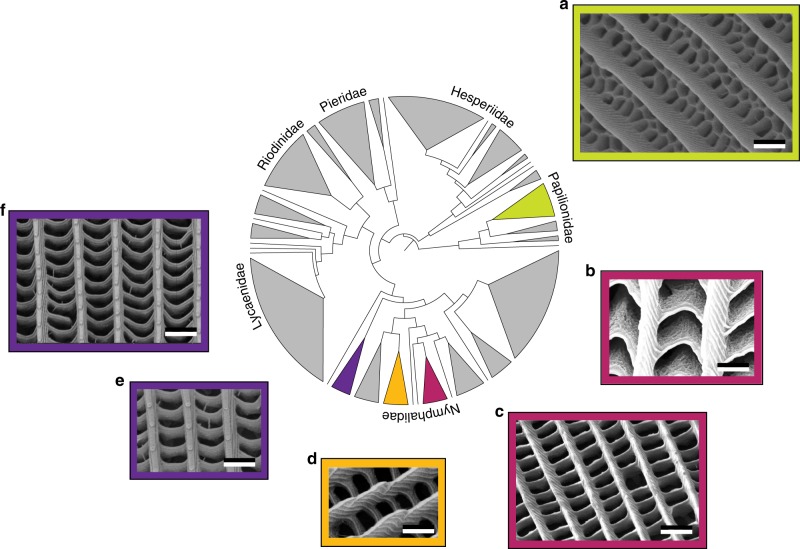
Ultra-black butterfly scales are widespread and morphologically diverse. A sub-family level phylogeny of butterflies adapted from ref. ^22^ showing the distribution of ultra-black coloration and SEMs of the underlying nanostructures. Note the variation in hole shape and size including a honeycomb (**a**), large and small rectangles (**c**–**f**), and chevron-shaped holes (**b**). Subfamilies shown in gray have no known ultra-black representatives. All scale bars are 1 µm. Color of frames corresponds to color of sub-family on the phylogeny. Additional images in Supplementary Fig. 1. Species: **a** *Trogonoptera brookiana* (male), **b** *E. chlorocroa*, **c** *C. antinoe*, **d** *H. doris*, **e** *E. dufresne*, **f** *E. klugi*.

Ultra-black materials depend ultimately on an absorbing pigment embedded in a complex ultrastructure. We first characterized the scale structure of 11 butterflies (seven ultra-black, four control) using scanning electron microscopy (SEM). All of the butterflies possessed scales with an upper lamina perforated by quasi-periodic holes (Fig. 2). There was considerable variation in the shape and size of the holes, with chevron-shaped holes in *Eunica chlorocroa*, 500 × 330 nm rectangular holes in *Catonephele antinoe, Catonephele numilia*, and *Heliconius doris*, and 750 × 500 nm rectangular holes in *Euploea dufresne* and *Euploea klugi* (Fig. 2 and Supplementary Fig. 2). Notably, none of the nymphalid butterflies (those in the subfamilies Biblidinae, Danainae, and Heliconinae) had a honeycomb structure analogous to the one found in papilionids. The lack of a honeycomb structure, coupled with the variation in the size and shape of the holes, suggest that ultra-blackness in butterflies is not dependent on a particular hole shape or size, although some of the intra-individual variation in hole shape likely helps increase absorption at non-normal incidence angles^14,15^.

Two structural features, steep longitudinal ridges and robust trabeculae connecting the upper and lower laminae, were consistently found in all of the ultra-black specimens. Control butterflies had larger holes and either lacked or showed significantly reduced trabeculae (Supplementary Figs. 1 and 2). The presence of the ridges and large trabeculae in evolutionarily distant ultra-black butterflies, and the lack of robust trabeculae in control butterflies, suggests both of these features are important for producing low reflectance. Remarkably, all of the butterflies that possessed these features retained their black color when coated with gold for SEM while those that did not became reflective (Supplementary Fig. 3).

### Finite-difference time-domain modeling

**Fig. 3:**
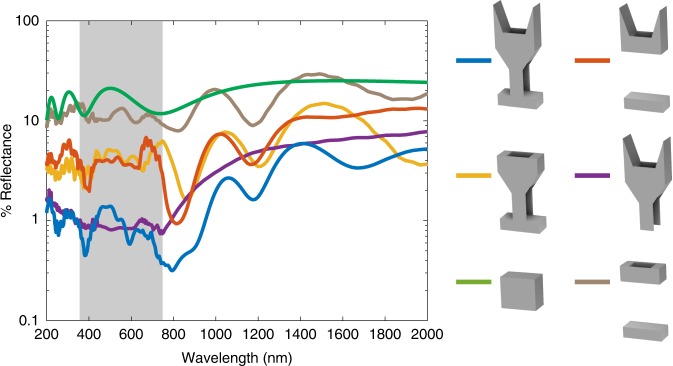
Removing conserved structural elements increases reflectance 3- to 16-fold. Reflectance of two overlapping scales backed by a 100% reflecting white surface simulated with the finite-difference time-domain method (unit cell for each simulation is shown). Included is the full-scale model (blue), scales with the trabeculae removed (red), with the ridges removed (yellow), with the basal lamina removed (purple), both ridges and trabeculae removed (brown), and a rectangular block made of the same volume of absorbing material as the full-scale (green). Full scales reflect 2.5–10% of the light reflected from a rectangular block of absorbing material. Removing the robust trabeculae or ridges increases reflectance by 3- to 16-fold, while the lamina has little effect on the reflectance. Curves are made by averaging five simulation runs with unit cell sizes spanning +/− 5% of the standard cell.

After identifying the geometrical components common to all ultra-black butterflies among our sampled species, we determined their relative contribution to reflectance reduction. As a scale could have low reflectance and be transparent (e.g., moth-eye structure) instead of black like the butterfly wings, we calculated the reflectance of two overlapping scales backed by 100% white surface to determine a measure of blackness. We used the finite-difference time-domain (FDTD)^16^ method to simulate the reflectance of a structure with rectangular 500 × 330 nm holes, the dimensions of the blackest butterfly we measured, *C. antinoe* (model parameters Supplementary Table 2; for schematic see Supplementary Fig. 4). We then ran the same simulation with the ridges, trabeculae, or basal lamina removed. The full butterfly scale model had a reflectance between 0.4 and 1.0% across the visible part of the spectrum (Fig. 3). This was 14–40 times lower than the simulated reflectance of two flat overlapping slabs on a 100% white background made from the same volume of absorbing material. Removing either the ridges or inner structure resulted in a three- to 16-fold increase in reflectance, and removing both increases reflectance by 7- to 28-fold. In contrast, removing the same volume of the absorbing material from a flat block only increases the reflectance by at most twofold (Supplementary Fig. 5). Changes in reflectance after removing the basal lamina were marginal. The increase in reflectance when removing the trabeculae or ridges is consistent with the hypothesis that these are key structural components for making an ultra-black structure.

**Fig. 4:**
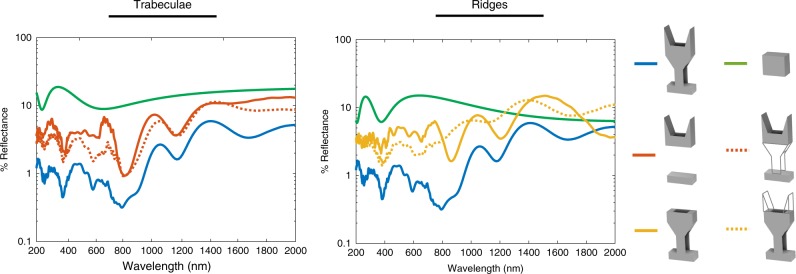
Decoupling the effects of structure and absorption on scale reflectance. (Left) Comparing simulations of scales with the trabeculae removed versus scales with non-absorbing trabeculae. Adding non-absorbing trabeculae to a scale that has none decreased reflectance between 5 and 70% in the visible range (350–700 nm). (Right) Comparing simulations of scales with the ridges removed versus scales with non-absorbing ridges. Non-absorbing ridges contribute a reduction in reflectance between 14 and 58% in the visible range (350–700 nm). For both the ridges and the trabeculae, the structure contributes more to reduced reflectance in the visible range.

To decouple the relative contributions of the structure to reflectance from the absorbing properties of the scale material, we simulated butterfly scales with either transparent ridges or transparent trabeculae while holding the real part of the refractive index constant. We found that the structure of the ridges alone decreases reflectance between 14 and 58% compared to a scale that has no ridges at all, and the structure of the trabeculae decreases reflectance between 5 and 70% compared to a scale that lacks them (Fig. 4). That is to say, these structural features alone significantly reduces reflectance, even when they do not directly contribute to absorption. It should also be noted that the reduction in reflection is more prominent in the visible range, indicating specialization for absorbing visible light.

**Fig. 5:**
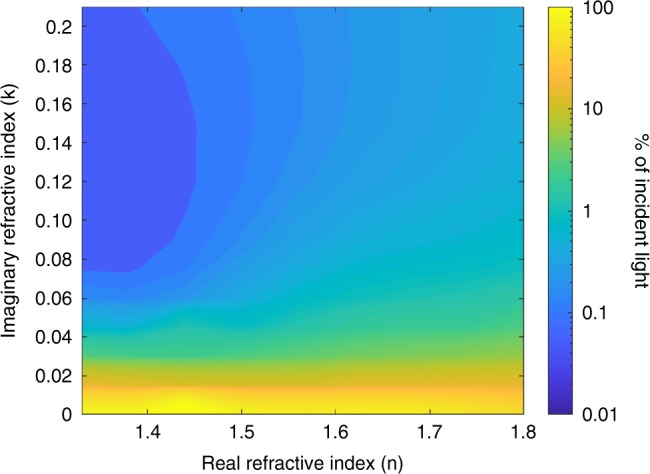
Reflectance is primarily driven by the imaginary part of the refractive index. Contour plot of the simulated reflectance at 550 nm from scales with 99 unique combinations of real and imaginary parts of the refractive index. Reflectance is considered to be the reflectance from two overlapping scales backed by a 100% reflecting white surface. Real part of the refractive index varies from water (*n* = 1.33) to melanin (*n* = 1.8), and the imaginary part varies from *k* = 0.0 (no absorption) to *k* = 0.20. Color indicates reflectance.

After characterizing the underlying structure, we next examined the absorbing pigment, melanin, to test whether its specific optical properties are critical in creating an ultra-black scale. Melanin has an unusually high real (*n*) and imaginary (*k*) refractive index for a biological material^17^. Using the morphological parameters and configuration as described above, we simulated the reflectance at 550 nm using 99 unique combinations of real and imaginary parts of the refractive index. We varied the real part of the refractive index from *n* = 1.33 (water) to *n* = 1.8 (melanin) and varied the imaginary part from *k* = 0.0 (no absorption) to *k* = 0.20 (Fig. 5). With no absorption, the reflectance from two scales overlaying a white background approaches 100%, but this decreases to 1% with *k* = 0.06 (the imaginary part of the refractive index of a black *Morpho didius* ground scale)^18^. The effect of the real refractive index is dominated by that of the imaginary refractive index until *k* > 0.06. For a scale with a high imaginary part (*k* > 0.10) the pattern reverses, and a high real part is primarily responsible for increases in reflectance. For example, when *k* = 0.15, the reflectance when *n* = 1.33 is 88% lower than when *n* = 1.8. When 0.06 < *k* < 0.10, the reflectance depends on both components of the refractive index. This suggests that melanin’s particular optical properties (high real and imaginary components of the refractive index) are not necessary to make ultra-black butterfly scales, only a strongly absorbing material—ideally one with a real part of the refractive index lower than that of melanin.

## Discussion

Our results demonstrate that butterflies have convergently evolved nanostructures that reflect as little as 0.06% of incident light at 90° incidence. Consistently, ultra-black butterflies have substantially larger trabeculae between the upper and lower scale lamina that increase the surface area for absorption by cuticular melanin. Both the ridges and trabeculae increase the absorption of the entire scale with their structure alone, even excluding the absorption of the individual component. This is consistent with a growing body of literature supporting sparse packing, high surface area, and strong absorption as the general design principles of natural ultra-black materials like bird of paradise feathers^10^ or synthetic ones such as carbon nanotube arrays^5,6^. Interestingly, in the butterflies, these principles are being applied in a layer that is only 1/5th the thickness of synthetic or other natural materials.

Similar to other work, we find that the nano-holes are crucial for reducing reflection by absorbing light that is channeled into the holes^7,9,13^. However, previous modeling of ultra-black butterfly scales has only included the ridges, holes, and upper and lower lamina of the scale, neglecting the trabeculae^13^. The trabeculae were found here to be the major structural difference between regular and ultra-black butterfly scales, and they contribute to much of the increase in absorption in our model. In addition, unlike prior work that found the ridges have an insignificant effect on broadband absorption^9^, we found support for a substantial role of the periodic ridges in reducing reflection that is on par with that of the trabeculae. These differences in the role of the ridges, however, may be the result of differences in scale geometry. Papilionid butterflies have two rows of quasi-periodic holes between ridges that increases the spacing between ridges to >1 μm, which may reduce the contribution of the ridges to absorption. Nymphalid butterflies, by contrast, typically have a ridge spacing within the wavelength range of visible light. Additionally, the diversity of hole shapes (chevrons, rectangles, and quasi-honeycombs) and sizes (ranging from 350 to 750 nm) suggest that enhanced absorption from resonance effects when the hole radius (*r*) ≈ wavelength (*λ*) may be either less important or a more flexible criterion than previously hypothesized^13^.

It is uncertain what evolutionary pressures have led to ultra-black coloration in butterflies. However, many of the species here display in open, sunny locations where a more reflective black material would produce significant specular reflections that would decrease the contrast of colored patches used in inter- and intraspecific signaling^19^. Indeed, black color is sexually dimorphic in *Trogonoptera* and *Catonephele* with males displaying ultra-black patches and females displaying a black with a higher reflectance^20,21^. Ultra-black wing patches frequently border brightly colored areas, as is found in in other ultra-black animals^10,11^, and our findings are consistent with the hypothesis that butterflies use these ultra-black patches to enhance the contrast of those colors in a signaling context^8^.

Further research is needed to understand the biological function of ultra-black scales in inter- and intraspecific interactions, as well as evaluate the suitability of butterfly scales for replication as a synthetic ultra-black material. Despite having a slightly higher reflectance than carbon nanotube alternatives, butterfly scales offer two key advantages for replication: they are thinner than known alternatives and they can be fabricated at lower temperatures via plasma-enhanced chemical vapor deposition^9^ instead of being grown from carbon nanotubes. These findings have important implications for the design of optical instrumentation, photovoltaic cells, and, if scaled up in size, radar absorbing materials.

## Methods

### Specimen acquisition

Five species of ultra-black papilionid butterflies (*Trogonoptera brookiana* male*, Parides iphidamus, Papilio bangui, P. oribaeus*, and *Troides helena*), one regular black papilionid specimen (*Trogonoptera brookiana* female*)* and seven species of nymphalid butterflies (*Euploea dufresne, Euploea klugi, Euploea midamus, Heliconius doris, Heliconius wallacei, Catonephele antinoe*, and *Eunica chlorocroa*) were obtained from Fred Nijhout’s collection at Duke University. The remaining specimens (*C. numilia* male and female, *Heliconius ismenius*) were acquired from the North Carolina Museum of Life and Science (Durham, NC, USA). All specimens were kept mounted in wooden insect boxes with naphthalene to prevent decay between spectrophotometer measurements and electron microscopy. Every specimen was chosen for minimal wear, as missing scales create small reflective spots on the wing. As we were interested in the structural components low reflectance, and not variation within a particular species, we chose one specimen per species.

### Reflectance measurements

Spectral reflectance from the butterfly wings was measure using a multi-channel spectroradiometer (R400-7 Ocean Optics Inc., Dunedin, FL, USA) coupled with a Xenon ultraviolet (UV)–visible (VIS) light source (PX-2 Ocean Optics) and fiber-optic back-reflectance probe (USB2000). All measurements were taken at 90° relative to the plane of the wing to capture the maximum specular reflectance and thus provide a conservative estimate of how black a material is. The measurements from 3 to 5 locations on the wing were averaged for each species. During measurements, the wings were placed on a Spectralon^TM^ block with 2% diffuse reflectance (Labsphere, North Sutton, NH, USA). Reflectance measurements were calibrated with the same 2% reflectance standard instead of a typical 99% reflectance standard. We used the black standard as opposed to a typical white standard because the reflectance of the wings was so low that the signal to noise ratio was small when calibrating with a white standard.

### Characterization of wing scales

Small sections from the wings of the ultra-black butterflies *Trogonoptera brookiana* (male), *Catonephele antinoe, Catonephele numilia* (male)*, Heliconius doris, Napeocles jucunda, Eunica chlorocroa*, and *Euploea dufresne, Euploea klugi*, were mounted on aluminum SEM stubs with copper tape and sputter coated with ~7.5 nm of gold (Denton Desk V; Denton Vacuum LLC, Moorestown, NJ, USA). Sections from regular black and dark brown butterflies *Trogonoptera brookiana* (female)*, C. numilia* (female)*, H. ismenius*, and *Euploea midamus* were mounted and coated using a similar protocol. We imaged the scales using an Apreo S scanning electron microscope (ThermoFisher Scientific, Waltham, MA, USA) at accelerating voltages of 1–5 kV and magnifications of ×512–×100,000.

### Finite-difference time-domain modeling

Three-dimensional simulations of the reflectance and transmittance of ultra-black butterfly scales were performed with a Python implementation of the freely available software package MIT Electromagnetic Equation Propagation (MEEP)^16^. MEEP implements the finite-difference time-domain (FDTD) modeling in which differential forms of Maxwell’s equations are solved on a discrete grid and electromagnetic fields are evolved in time, step-by-step. We first verified our model by simulating the reflectance of a clear and an absorbing slab. All simulations were performed with a unit cell geometry and periodic boundary conditions in the z- and y-directions with perfectly matched layer (PML) absorbers in the x-direction (schematic of the unit cell: Supplementary Fig. 3). The source was a Gaussian pulsed plane wave traveling in the x-direction with a central frequency corresponding to a wavelength of 360 nm and covering the range 200–2000 nm. The linear resolution was set to 20 nm to allow for at least ten sampling points per wavelength. All calculations were performed in the far field (>4 wavelengths from the scale).

We created a custom geometry that consisted of one hole through the upper lamina, the crossribs, half of each scale ridge, and a lower lamina, that represented the unit cell of an ultra-black butterfly scale. We considered the standard unit cell to have a 500 × 300 nm hole, 600 nm ridges, and 1200 nm spacing between the upper and lower lamina (values derived from *C. antinoe*, the blackest butterfly considered here). The standard cell was simulated with a complex refractive index of 1.56*n* ± 0.06i, the reported value for a black *Morpho* ground scale^18^. We used a fixed value for the refractive index because the exact dispersion of ultra-black scales is currently unknown, and the models are intended to identify important geometric features, not to exactly recreate the reflectance of a butterfly scale. Melanin was assumed to be evenly distributed throughout the structure as it appears to be in other ultra-black butterflies^8^.

To determine the effects of specific structural features on the blackness of the scale, we systematically removed parts of the structure and repeated the simulation performed for the standard cell. As some modifications reduce reflectance (*R*) but greatly increase transmission (*T*), which ultimately will be reflected back by the wing substrate or another scale, we compared the reflectance from two scales backed by a perfect reflector. This can be approximated by the expression *R* + *R*(*T*^2^) + *T*^4^ (this expression was also used to calculate the values seen in Figs. 3 and 4). The structural modifications with the greatest effect on reflectance: removing the robust trabeculae, removing the scale ridges, and removing the lower lamina, are included here. All results reported in Fig. 3 are the result of averaging five simulations where the unit cell size is varied from 95% of the size of the original model to 105% of the size of the original model. This was done to eliminate any wavelength-specific resonant effects that may occur from having a perfectly periodic structure that does not reflect a real biological material.

As a means of decoupling the effect of the structure’s effect on absorption from the direct absorption of the material making up the ridges or trabeculae, we simulated two additional sets of scales with either transparent ridges or transparent trabeculae. We did this by keeping the real part of the refractive index the same as the rest of the scale and setting the imaginary part to zero just for the component of interest.

To investigate whether the specific optical properties of melanin—high real and imaginary parts of the refractive index—are necessary to make this structure function, we decoupled the real and imaginary parts of the refractive index. We simulated the reflectance of scales with 99 unique combinations of real (*n*) and imaginary (*k*) parts at 550 nm (a relevant wavelength of light in forest light that has been filtered by a canopy). We simulated *n* = 1.33, 1.38, 1.44, 1.50, 1.56, 1.62, 1.68, 1.74, 1.80 each with the following values for imaginary part of the refractive index: *k* = 0.0, 0.015, 0.03, 0.045, 0.06, 0.075, 0.09, 0.12, 0.15, 0.18, 0.20. The reflection from two wings backed by a reflector was plotted as a contour plot in Matlab using linear interpolation (Fig. 4).

## Supplementary information

Supplementary Information

## Data Availability

The authors declare that the data supporting the findings of this study are available within the paper and the supplemental information files. The source data underlying Figs. 1, 3–5 are provided as a Source Data file. All FDTD simulations were performed using the open-source software package pyMEEP.

## Source data

Source Data file

## Code availability

The code used in this study is available from the authors by request.
