# Supplementary material for: Diverse nanostructures underlie thin ultra-black scales in butterflies
Source: Nat Commun. 2020 Mar 10;11:1294. doi: 10.1038/s41467-020-15033-1 (PMC7064527; doi:10.1038/s41467-020-15033-1)
Supplement: Supplementary file 1 — Supplementary Information [file 41467_2020_15033_MOESM1_ESM.pdf]

Supplementary Materials for

Diverse nanostructures underlie thin ultra-black scales in butterflies

Alexander L. Davis[1*], H. Frederik Nijhout[1], Sönke Johnsen[1]
Correspondence to: alexander96davis@gmail.com

This PDF file includes:

Supplementary Tables 1,2
Supplementary Figures 1-5

Other Supplementary Materials for this manuscript include the following:

Source Data file

Supplementary Table 1. Specimen list. List of specimens with reflectance of the black patches measured in this study (n = 16). Hole shape and wing color are indicated in the fourth and fifth column.

Family	Subfamily	Species	Color	Hole Shape
**Nymphalidae**				
	Biblidinae			
		Catonephele antinoe	**Ultra-black**	Rectangular
		Catonephele numilia (f)	Black	Rectangular
		Catonephele numilia (m)	**Ultra-black**	Rectangular
		Eunica chlorocroa	**Ultra-black**	Chevron
	Danainae			
		Euploea dufresne	**Ultra-black**	Rectangular
		Euploea klugi	**Ultra-black**	Rectangular
		Euploea midamus	Matte Brown	Crescent
	Heliconinae			
		Heliconius doris	**Ultra-black**	Rectangular
		Heliconius ismenius	Black	Rectangular
		Heliconius wallacei	**Ultra-black**	
**Papilionidae**				
	Papilioninae			
		Trogonoptera brookiana (f)	Brown	Honeycomb
		Trogonoptera brookiana (m)	**Ultra-black**	Honeycomb
		Troides helena	**Ultra-black**	Honeycomb
		Papilio bangui	**Ultra-black**	
		Papilio iphidamus	**Ultra-black**	
		Papilio oribaeus	**Ultra-black**	

Supplementary Table 2. Simulation model parameters. Size parameters used for the FDTD simulations. Parameters were taken from *C. antinoe*, our blackest species.

Model Parameter	Size (nm)
Hole width (long axis)	500
Hole width (short axis)	330
Inter-lamina distance	1200
Ridge height	600
Upper lamina thickness	200
Crossrib thickness	80

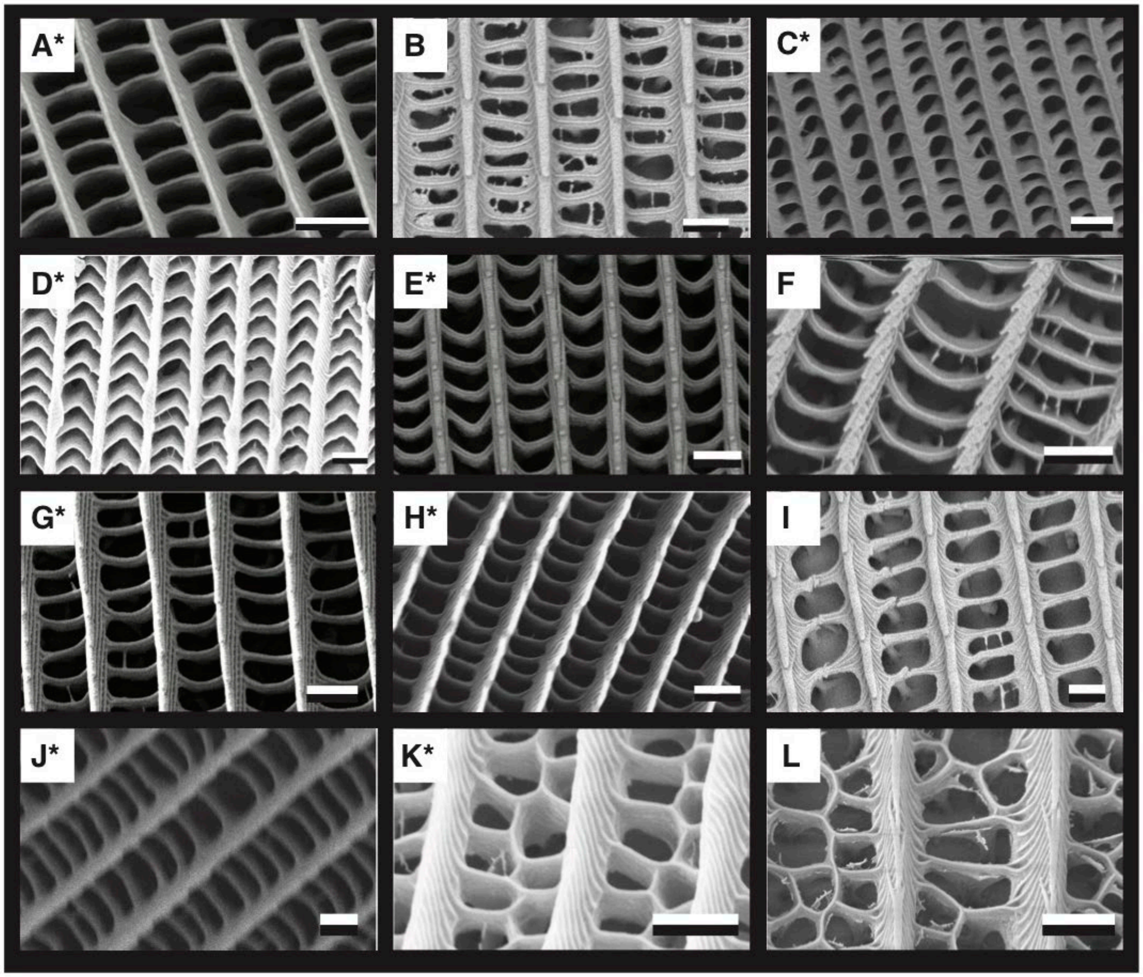

Supplementary Figure 1. Diversity of hole shapes and sizes.

(**A**) *Catonephele antinoe* **(B)** *Catonephele numilia* female **(C)** *Catonephele numilia* male **(D)** *Eunica chlorocroa* **(E)** *Euploea dufresne* **(F)** *Euploea midamus* **(G)** *Euploea klugi* **(H)** *Heliconius doris* **(I)** *Heliconius ismenius* **(J)** *Napeocles jucunda* **(K)** *Trogonoptera brookiana* male **(L)** *Trogonoptera brookiana* female; All species possess periodic holes bordered by long ridges, but the holes are smaller in ultra-black species compared to closely related black or brown species. All scale bars are 1µm. *denotes ultra-black species.

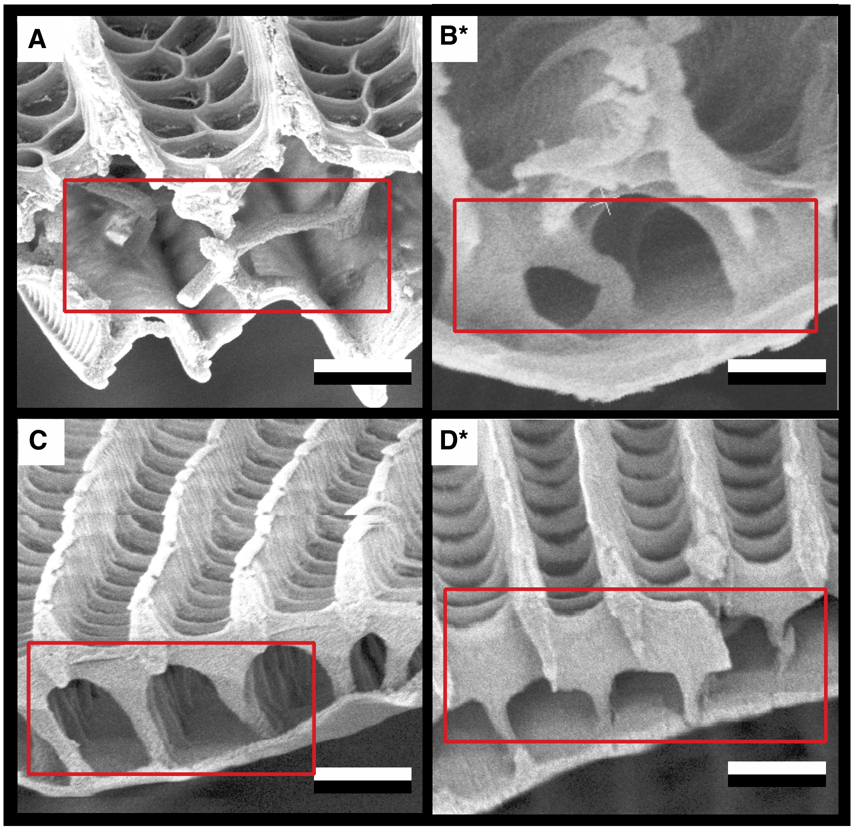

Supplementary Figure 2. Trabeculae are larger in ultra-black male butterflies compared to regular black female butterflies.

(A) *Trogonoptera brookiana* female **(B)** *Trogonoptera brookiana* male **(C)** *Catonephele numilia* female **(D)** *Catonephele numilia* male. The trabeculae (outlined in the red box) is larger in ultra-black male butterflies than in regular dark brown/black females of the same species.

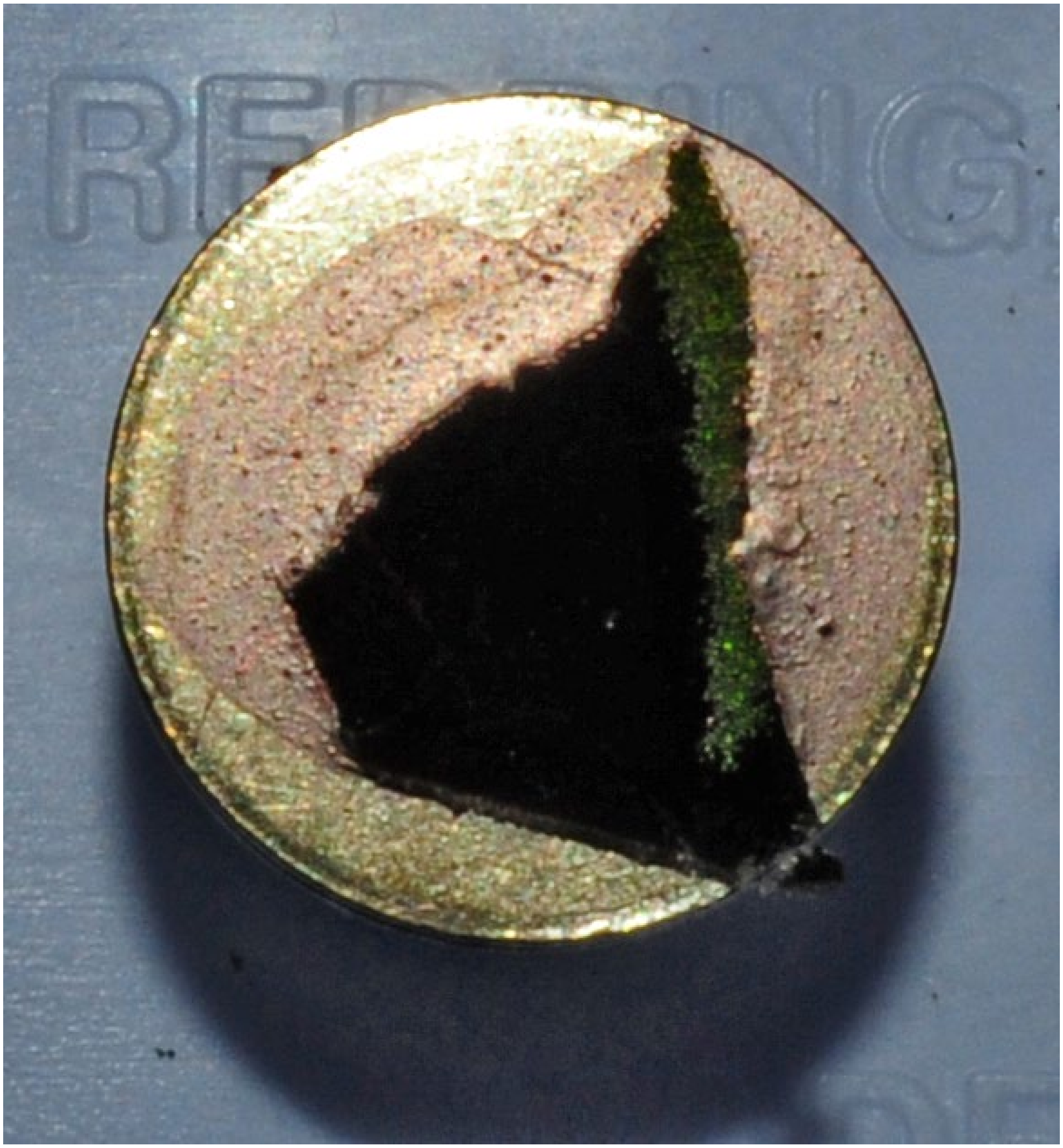

Supplementary Figure 3. Piece of butterfly wing sputter coated with gold. Ultra-black butterfly wings are still black when coated with gold, indicating that there is a structural component to the absorption.

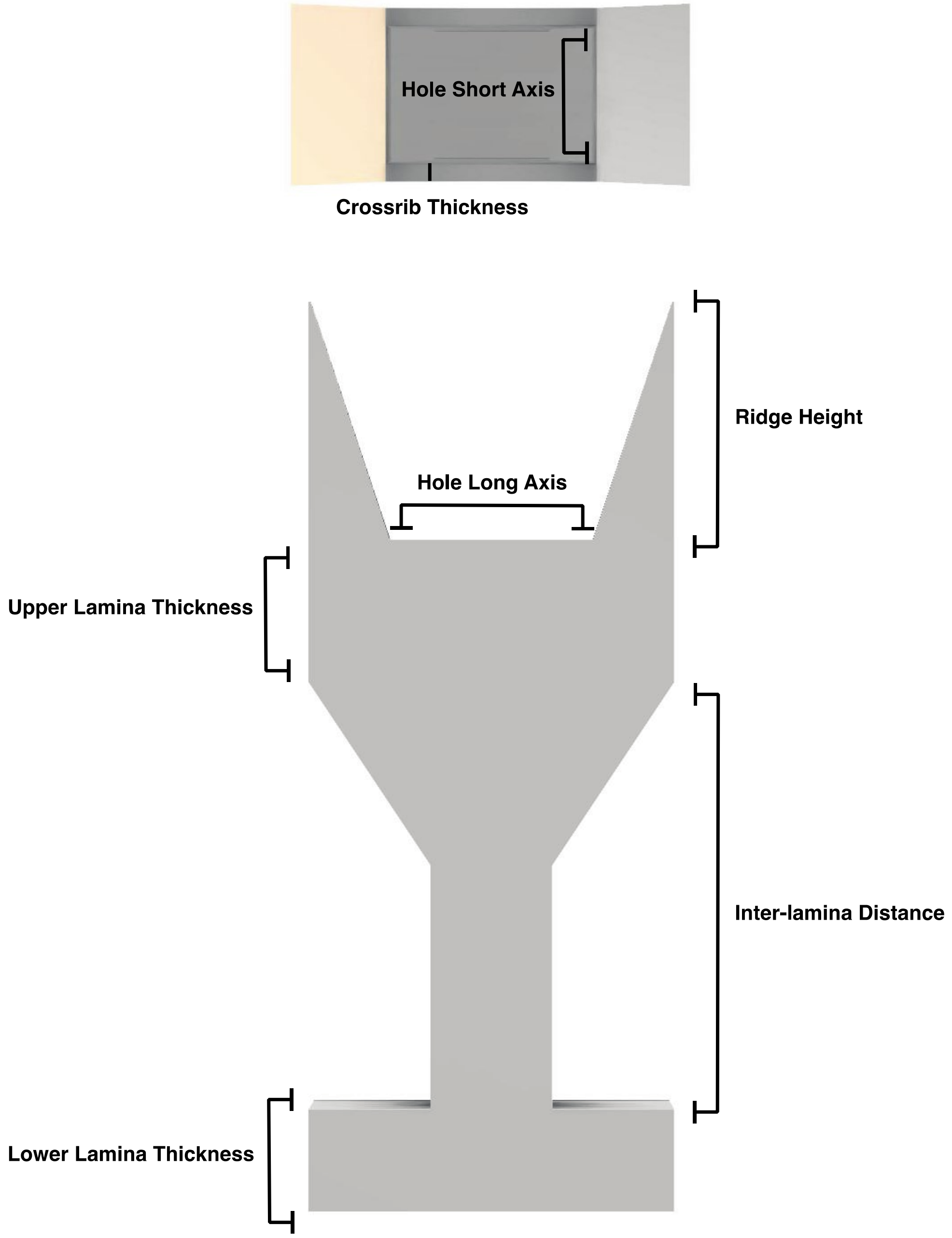

Supplementary Figure 4. Unit Cell Diagram. Schematic diagram of the unit cell that was used for FDTD simulations. This unit cell was simulated with periodic boundaries to form a semi-infinite sheet of repeating cells.

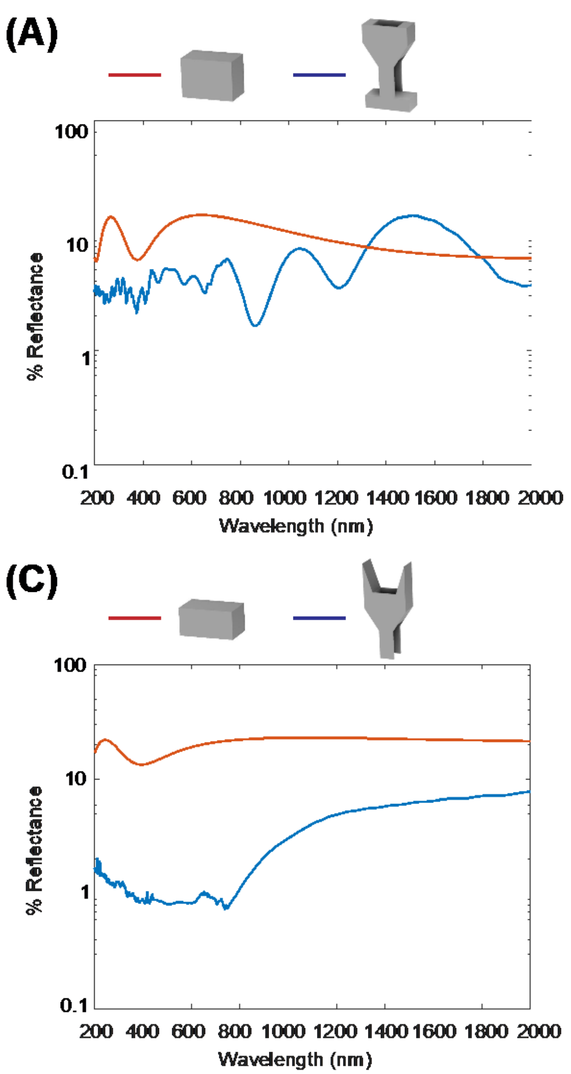

Supplementary Figure 5. Simulated reflectance of butterfly geometries and blocks consisting of an equal volume of absorbing material.
